# Lithium and Therapeutic Targeting of GSK-3

**DOI:** 10.3390/cells10020255

**Published:** 2021-01-28

**Authors:** Melinda E. Snitow, Rahul S. Bhansali, Peter S. Klein

**Affiliations:** Department of Medicine, Perelman School of Medicine, University of Pennsylvania, 3400 Spruce St., Philadelphia, PA 19104, USA; snitow@pennmedicine.upenn.edu (M.E.S.); Rahul.Bhansali@Pennmedicine.upenn.edu (R.S.B.)

**Keywords:** lithium, GSK-3, Wnt, bipolar disorder, cancer, coronavirus, severe acute respiratory syndrome (SARS), nucleocapsid

## Abstract

Lithium salts have been in the therapeutic toolbox for better or worse since the 19th century, with purported benefit in gout, hangover, insomnia, and early suggestions that lithium improved psychiatric disorders. However, the remarkable effects of lithium reported by John Cade and subsequently by Mogens Schou revolutionized the treatment of bipolar disorder. The known molecular targets of lithium are surprisingly few and include the signaling kinase glycogen synthase kinase-3 (GSK-3), a group of structurally related phosphomonoesterases that includes inositol monophosphatases, and phosphoglucomutase. Here we present a brief history of the therapeutic uses of lithium and then focus on GSK-3 as a therapeutic target in diverse diseases, including bipolar disorder, cancer, and coronavirus infections.

## 1. History of Lithium Treatment

Lithium was discovered as a new element (atomic number 3) in 1817. Lithium is an alkali metal, the lightest solid element, and a monovalent cation in solution, used therapeutically as lithium carbonate (most common), acetate, citrate, chloride, or sulfate salts. Lithium salts first gained pharmacological popularity in the 19th century as treatment for gout and other “gouty” conditions thought to be caused by excessive uric acid, as lithium carbonate dissolved uric acid in vitro. By 1859, Sir Alfred Baring Garrod’s work on gout included his recommendation for lithium to treat “brain gout”, including mania. In 1871, lithium bromide was used to treat acute mania by William Hammond at Bellevue Hospital. Danish psychiatrists Carl and Frederik Lange recommended lithium carbonate as maintenance therapy to treat periodic depression, which they believed to be caused by uric acid. The “uric acid diathesis” hypothesis of disease was largely abandoned in the early 1900s [1,2,3,4].

Lithia water and lithium tablets were popular patent medicines and health supplements in the late 19th and early 20th centuries purported to prevent or treat uric acid-induced ailments. The carbonated beverage now known as 7Up was originally sold as “Bib-Label Lithiated Lemon-Lime Soda” and was marketed as a hangover cure. Lithium toxicity was described in several studies, but lithium salts remained popular in the United States until patent medicines were restricted by the FDA. In 1948, lithium chloride (LiCl) was marketed as a table salt substitute for low-sodium diets, resulting in multiple reports of lithium toxicity, including fatal cases by 1949 [2,5,6].

In 1949, Australian psychiatrist John Cade reported successfully using lithium citrate to treat patients institutionalized with mania after observing a sedative effect of lithium on guinea pigs in uric acid experiments and after assessing the safety of lithium by self-administration [7]. Cade demonstrated remarkable improvement in multiple patients who had been institutionalized for years and also showed that patients reverted to mania when lithium was discontinued. While some have questioned whether Cade was aware of prior work with lithium in bipolar disorder patients [8], his landmark paper was confirmed in randomized controlled clinical trial led by Mogens Schou in Denmark in 1954 [9] and by others. Cade’s work therefore catalyzed the modern age of lithium therapy for bipolar disorder and stands as one of the earliest papers on rational medicinal therapy of major psychiatric disorders. Longitudinal studies by Schou and colleagues also demonstrated the prophylactic effect of lithium as a mood stabilizer to prevent recurrent manic and depressive episodes. The development of a simple assay for plasma lithium levels made it possible to establish a therapeutic range for lithium. This revealed that lithium has a narrow therapeutic window. The US FDA approved lithium for the treatment of mania in 1970, becoming the 50th country to do so, and approved lithium for maintenance therapy in 1974 [2,10,11].

## 2. Direct Targets of Lithium

The direct targets of lithium in psychiatry are unknown, but basic science research gives rise to several viable candidate targets. While lithium induces wide-ranging physiological and developmental effects, lithium directly inhibits few known targets. Establishing which of these targets is responsible for a given effect of lithium can be challenging, especially for the therapeutic effects in humans. Criteria for validating direct targets of lithium have been previously discussed [12,13]. Briefly, evidence to support a putative target of lithium includes (1) inhibition of the target enzyme at therapeutically relevant concentrations of lithium in vitro and in vivo, (2) structurally distinct inhibitors of the proposed target mimic the effect of lithium, (3) genetic loss-of-function of the proposed target mimics the effect of lithium, and (4) the effect of lithium is reversed by restoring target function or a downstream effector.

Direct targets of lithium include inositol monophosphatase [14] and structurally related, magnesium-dependent phosphomonoesterases. These share a metal ion binding consensus sequence and include inositol polyphosphate 1-phosphatase (IPPase), fructose 1,6-bisphosphatase (FBPase), and bisphosphate 3′ nucleotidases (BPntase) [12,15,16]. Phosphoglucomutase (PGM), which catalyzes the interconversion of glucose-1-phosphate (G1P) to glucose-6-phosphate (G6P), is structurally unrelated to these phosphomonoesterases but is also magnesium-dependent and directly inhibited by lithium [17]. Additionally, glycogen synthase kinase-3 (GSK-3), a CMGC protein kinase family member most closely related to cyclin dependent kinases (CDKs), is also inhibited by lithium [18].

While these three classes of lithium targets are structurally distinct, all are magnesium dependent, and in each case, lithium, which has an ionic radius similar to magnesium, competes for a magnesium binding site [15,16,19,20]. However, most magnesium dependent enzymes, including almost all protein kinases that have been tested, are not sensitive to lithium [18,21,22]. Furthermore, it is curious that all of the known targets of lithium are closely linked to the regulation of glucose or phospho-glucose levels. PGM isomerizes G6P to G1P; FBPase is required for gluconeogenesis; IMP is generated from isomerization of G6P; and GSK-3 suppresses incorporation of glucose into glycogen. The sensitivity of these enzymes to lithium is observed in organisms from protozoans to mammals.

### Evidence Supporting GSK-3 as a Lithium Target

Lithium salts have been widely used experimentally to disrupt cell fate specification in diverse organisms from protozoans to vertebrates. Development of the slime mold *Dictyostelium discoideum* is sensitive to lithium: Upon starvation these free living amoeboid cells form multicellular aggregates comprising stalk and spore cells. Lithium exposure prior to aggregation inhibits spore cell fate and promotes stalk cell fate [23,24]. Lithium also alters cell fate in sea urchin embryos, diverting animal cells to a vegetal fate in a process referred to as vegetalization [25,26]. Lithium also dorsalizes vertebrate embryos including *Xenopus* and zebrafish, expanding dorsal mesoderm and inducing a second dorsal axis [27,28,29]. Lithium also alters cell fate in the developing mouse kidney [30] and affects the development of multiple other organisms.

Lithium also stimulates glycogen synthesis in muscle, hepatocytes, and adipocytes by increasing the activity of Glycogen Synthase (GS), enhancing glucose uptake and storage and mimicking insulin action, although the direct target for these effects was not identified in those early studies [31,32,33,34,35].

Each of these developmental and metabolic effects of lithium is mimicked by inhibition or genetic loss of glycogen synthase kinase-3 (GSK-3). GSK-3 was so named because it phosphorylates and inhibits GS; thus the effects of lithium on insulin signaling and glycogen synthesis can be explained by inhibition of GSK-3, as demonstrated later [34]. Similarly, inhibition of GSK-3 offered a compelling explanation for the developmental effects of lithium. Knockout of *gska* in *Dictyostelium* caused expansion of stalk cell fate at the expense of spore cells [36] that was remarkably similar to the effect of lithium described by Maeda [24]. Furthermore, dominant negative forms of *Gsk3b* mimic the effect of lithium in *Xenopus* [37,38,39]. In the context of vertebrate development, GSK-3 functions as an antagonist of canonical Wnt signaling [40,41,42], and therefore lithium, as a GSK-3 inhibitor, is predicted to activate Wnt signaling. Indeed, Wnt signaling had recently been shown to recapitulate lithium phenotypes in *Xenopus* embryos, where Wnt activation also induces axis duplication [43].

Recognition that the metabolic and developmental effects of lithium parallel the effects of GSK-3 inhibition in these settings led to the hypothesis that lithium acts through direct inhibition of GSK-3, and this was demonstrated through in vitro assays using mammalian GSK-3β [18]. Subsequent work confirmed that lithium inhibits GSK-3 from mammals, amphibians, *Drosophila*, and *Dictyostelium* [22,44]. Lithium competes for a low K_m_ magnesium binding site in GSK-3 with a K_i_ ~1–2 mM [19,20]. Other monovalent cations do not mimic this effect on GSK-3 activity, and the effect of lithium is independent of the anion species [18,19,20,22]. Furthermore, lithium does not significantly inhibit multiple other protein kinases, demonstrating relative selectivity for GSK-3 [18,21].

## 3. GSK-3 Function

**Figure 1:**
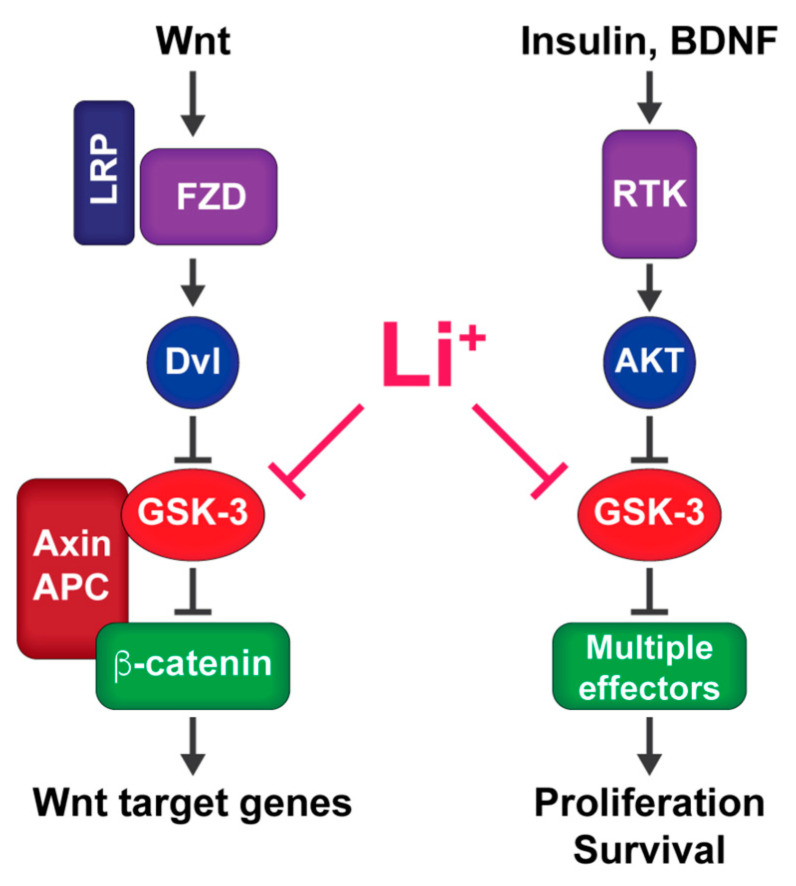
Signaling pathways that inhibit GSK-3: Wnt signaling (left) inhibits GSK-3 through an allosteric mechanism to stabilize β-catenin and activate Wnt target genes. Insulin, BDNF, and other receptor tyrosine kinase (RTK) ligands that act through PI3 kinase to activate AKT inhibit GSK-3 through N-terminal phosphorylation to modulate diverse downstream pathways. Lithium mimics both types of signaling pathways by directly inhibiting GSK-3.

GSK-3 is a serine/threonine kinase expressed in most vertebrates as two highly similar and mostly redundant isoforms, *GSK3A* and *GSKB*, which encode the proteins GSK-3α and GSK-3β [45,46] (for simplicity, we will refer to both as GSK-3 unless otherwise specified). GSK-3 is ubiquitously expressed in mammalian tissue, and is evolutionarily conserved across slime molds, yeast, plants, and animals [12,47,48]. GSK-3 is constitutively active and is negatively regulated by phosphorylation on serine-21 (GSK-3α) and serine-9 (GSK-3β) primarily through activation of Akt (aka PKB), but also through growth factor stimulation of mitogen activated protein kinases (MAPKs), mechanistic target of rapamycin (mTOR), protein kinase A (PKA), and protein kinase C (PKC) [40,49]. GSK-3 is also constitutively phosphorylated at a conserved tyrosine within the activation loop (tyrosine-279 in GSK-3α and tyr-216 in GSK-3β), which may facilitate substrate binding [50,51,52]. GSK-3 is inhibited by Wnt signaling independently of GSK-3 Ser21/9 phosphorylation [53,54,55] (Figure 1).

GSK-3 was first identified among several protein kinases that phosphorylate glycogen synthase [46], but since then many other direct targets of GSK-3 have been identified, including the Wnt effector protein β-catenin, other transcription factors, multiple RNA splicing factors, regulators of translation, protein kinases, and cytoskeletal proteins [40,56,57]. GSK-3 target phosphorylation frequently impairs the activity or stability of its targets, including glycogen synthase and β-catenin [56], although GSK-3 can also stabilize substrates such as the nuclear hormone receptor Rev-erbα [58] and enhance the function of targets including the tuberous sclerosis complex 2 protein [59].

Multisite phosphorylation of GS by GSK-3 established a paradigm for many GSK-3 substrates in which GSK-3 depends on prior phosphorylation at the +4 position [60,61]. This “priming” phosphorylation enhances GSK-3 phosphorylation of a serine or threonine 4 residues to the N terminal side of the priming site. GSK-3 substrates such as GS and β-catenin frequently have multiple serines or threonines spaced 4 residues apart, allowing processive phosphorylation in the C to N terminal direction. Phosphorylation of N terminal serines in GSK-3 itself creates a pseudosubstrate that inhibits activity toward primed substrates [61]. However, many GSK-3 substrates, including Inhibitor-2 (I-2) and Tau protein, do not contain +4 priming sites [56,57], and the mechanisms of substrate recognition in these cases are less well characterized.

GSK-3 inhibition by Wnt signaling does not involve N-terminal phosphorylation [53,54,55]. Several non-exclusive mechanisms for Wnt-mediated inhibition of GSK-3 have been proposed, including inhibition by the phosphorylated C-terminus of the LRP5/6 co-receptor [62] and dissociation of APC, which enhances GSK-3 activity, from the Axin scaffold [63,64,65]. On a longer time scale, Wnt signaling drives sequestration of GSK-3 in the Axin complex into distinct intracellular pools termed multivesicular bodies, which protects β-catenin and other GSK-3 substrates from phosphorylation and degradation [66]. These mechanisms are not mutually exclusive but they are independent of Akt signaling and N-terminal phosphorylation.

## 4. GSK-3 and Human Disease

As outlined above, GSK-3 phosphorylates multiple substrates involved in critical cellular processes aside from its classical role in glycogen metabolism [40,56,57,67]. Many of these processes, such as autophagy, cell survival/differentiation, and cell cycle regulation, have clear implications in the pathogenesis of human disease. This has consequently led to substantial interest in better understanding the role of GSK-3 and related pathways as therapeutic targets in these disease models. Below, we will review known roles of GSK-3 in bipolar disorder, cancer, neurodegenerative diseases, and virally mediated diseases with an emphasis on targetable pathways.

### 4.1. Bipolar Disorder and Lithium

Bipolar disorder is a major psychiatric disorder associated with episodes of hyperactivity, elevated mood, and psychosis (mania and hypomania), periods of major depression, and a substantial risk of suicide [68]. Type I bipolar disorder is defined by at least one episode of mania and type II bipolar disorder is defined by episodes of both hypomania and major depression. The incidence of bipolar disorder is estimated to be 1–2% worldwide. Lithium is a first-line therapy for bipolar disorder, but the therapeutic window between the effective and toxic doses is narrow [68]. In addition to functioning as a mood stabilizer, lithium also reduces the risk of suicide in affective disorder patients, either as a monotherapy or adjunctive therapy. Lithium is more effective at suicide prevention than other mood stabilizers and antidepressants, although the mechanism is unknown [10,69,70]. Lithium is also an effective adjunctive therapy for treatment resistant depression, especially in combination with tricyclic antidepressants [71,72].

In addition to directly inhibiting GSK-3 enzymatic action [18,22], lithium also inhibits GSK-3 indirectly by increasing inhibitory Ser21/9 phosphorylation [73,74]. Another mood stabilizer (valproic acid), antidepressants (including the rapid-acting antidepressant ketamine), and antipsychotics also inhibit GSK-3 indirectly through Ser21/9 phosphorylation, suggesting a common mechanism involving GSK-3 [74,75,76,77,78].

The hypothesis that GSK-3 is the therapeutic target of lithium is supported by animal behavior studies using genetic manipulation of GSK-3. *Gsk3b* haploinsufficiency mimics lithium treatment in mouse behavioral tests that are responsive to lithium [79], while *Gsk3b* overexpression in the CNS blunts the response to lithium without affecting the animal’s health or activity levels [13], demonstrating a specific role for GSK-3 in lithium-responsive behavior. Furthermore, diverse small molecule inhibitors of GSK-3 also mimic the behavioral effects of lithium in mice [79,80,81,82,83], providing compelling support for GSK-3 as a critical lithium target in mouse behaviors. Conversely, a competing hypothesis that lithium acts therapeutically through IMPase inhibition to deplete inositol is not supported by animal behavior in genetic models of inositol depletion. Genetically reducing inositol levels to a greater extent than lithium did not phenocopy lithium treatment (or *Gsk3* haploinsufficiency) in mouse behavior [84], nor did depletion of inositol affect phosphatidylinositol abundance, arguing against this hypothesis [85,86,87]. Therefore, GSK-3 remains the best-supported direct target of lithium action in behavior.

**Figure 2:**
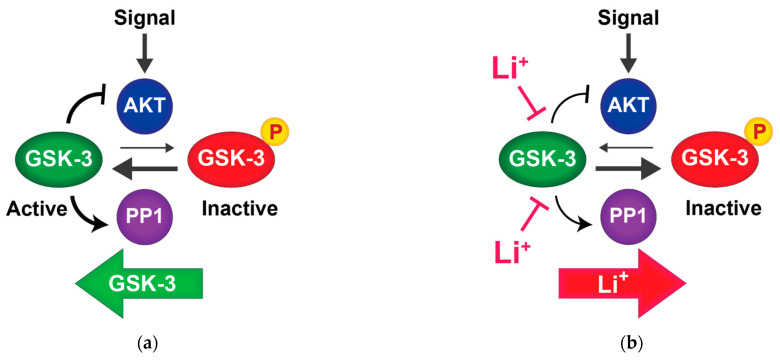
Positive feedback circuits regulate GSK-3: (**a**) GSK-3 is phosphorylated and inhibited by AKT in response to upstream signals. PP1 dephosphorylates and activates GSK-3. GSK-3 inhibits AKT and activates PP1, thereby enhancing its own activity; (**b**) lithium inhibits GSK-3 directly and disrupts both feedback circuits. Disruption of these feedback circuits by lithium may enhance the response to endogenous ligands that signal through AKT.

While GSK-3 is a promising target of mood stabilizers and other psychotropic drugs, it is unclear how its inhibition can encompass anti-manic, antidepressant, and antipsychotic effects. We propose a model in which mood stabilizers function through GSK-3 inhibition to re-sensitize cells to endogenous extracellular signals (Figure 2). Extracellular signals transduced through Akt (e.g., neurotrophins and neurotransmitters) lead to phosphorylation and inactivation GSK-3. However, GSK-3 opposes its own phosphorylation through at least two mechanisms. (1) GSK-3 inhibits Akt by binding to and stabilizing a β-arrestin complex that brings Akt into contact with its deactivating phosphatase PP2A. Inactivation of Akt thereby prevents phosphorylation and inhibition of GSK-3. Lithium or other GSK-3 inhibitors disrupt this complex [13,88], allowing Akt to remain active and phosphorylate GSK-3. (2) GSK-3 also maintains its own dephosphorylation by activating protein phosphatase 1 (PP1), which is inhibited by I-2; GSK-3 phosphorylation of I-2 causes it to dissociate from PP1, which then dephosphorylates GSK-3, restoring GSK-3 activity. Lithium or other GSK-3 inhibitors prevent PP1 from dephosphorylating GSK-3 in an I-2 dependent manner [73].

Therefore, pharmacological GSK-3 inhibition interrupts both autoregulatory feedback loops that drive GSK-3 to maintain an active state [13,73]. We hypothesize that pharmacological inhibition of GSK-3 lowers the threshold for endogenous signals to inactivate cellular pools of GSK-3, allowing weak or transient signals to effect a stable response through amplification of GSK-3 inactivation. This mechanism would allow lithium to sensitize cells to endogenous neurotransmitters or other signals that may be reduced in bipolar disorder and could explain why lithium is also effective in treatment resistant depression when used as an adjunct to antidepressants.

### 4.2. Neurodegenerative Disease

GSK-3 has well documented roles in neurodegenerative diseases, established through extensive mechanistic studies of GSK-3 in neurological models. Below, we provide a brief overview of the literature regarding GSK-3, neurodegenerative disease, and therapeutic insights.

#### 4.2.1. Alzheimer’s Disease and Related Tauopathies

Alzheimer’s disease (AD) is the most common form of neurodegenerative disease with increasing prevalence in an aging population [89]. Two pathologic findings characterize AD, neurofibrillary tangles (NFTs) and amyloid plaques, and both are promoted by GSK-3. NFTs are intracellular deposits of tau protein, a microtubule stabilizer that is regulated by phosphorylation; pathologic hyperphosphorylation of tau disrupts microtubules and promotes development of NFTs [90]. GSK-3 phosphorylates tau in vitro and in vivo at several residues, which can accelerate aberrant tau aggregation and resultant neurodegenerative phenotypes [91,92,93,94,95,96,97,98]. Second, β-amyloid (Aβ) peptides (primarily Aβ_40_ and Aβ_42_) accumulate in extracellular amyloid plaques. Plaque formation precedes tau pathology in AD and occurs through cleavage of the amyloid precursor protein (APP) into Aβ by β- and γ-secretases [99]. APP processing is also regulated by GSK-3. Both APP and presenilin-1 (PS1), which is part of the γ-secretase complex, are mutated in familial forms of AD, leading to increased accumulation of the more pathogenic Aβ_42_ peptide. Both APP and PS1 are GSK-3 substrates [100,101,102,103,104,105] and inhibition or knockdown of GSK-3 impairs APP processing, reducing generation of Aβ_40/42_ in mouse brain and cell culture models [94,105,106,107,108,109,110,111]. The requirement for GSK-3 in APP processing was challenged by a group that knocked out either *Gsk3a* or *Gsk3b* in mice and observed no effect on Aβ levels [112]. However, this report involved single gene knockouts only, overlooking the important and well established observation that the two genes are redundant in most functions [53], so that a single gene knockout may not affect APP processing in cells that express both genes equally. Furthermore, multiple structurally diverse GSK-3 inhibitors, including lithium, Tideglusib, kenpaullone, bisindolylmaleimide-I, FRAT peptide, and kinase-dead GSK-3 also impair APP processing [94,105,106,107,108,109,110,111], providing compelling support that GSK-3 facilitates APP processing. Moreover, Aβ aggregates activate GSK-3 and cause tau hyperphosphorylation [113,114,115]. This may form a feedback loop by which Aβ activates GSK-3, which then alters APP cleavage, resulting in more Aβ formation. These data suggest GSK-3 inhibition may have a role in slowing progression of AD, which is further supported by clinical studies described below.

As GSK-3 plays roles in both NFT formation and APP processing, GSK-3 has been a focus of extensive study for pharmacological interventions in AD. Inhibition with lithium and other agents in preclinical studies reduces Aβ mediated neurotoxicity, improves behavioral phenotypes, and rescues neuronal loss [105,116,117,118,119]. In addition, retrospective studies, meta-analyses, and a randomized controlled trial with patients with mild cognitive impairment showed that lithium prevented cognitive decline in patients with mild cognitive impairment when compared to matched patients not taking lithium [120,121,122]. Unfortunately, a phase II trial with the allosteric GSK-3 inhibitor Tideglusib did not achieve study endpoints for slowing cognitive decline [123] and studies with the GSK-3 inhibitors AZD2558 and AZD1080 were halted for intolerable safety profiles [124]. Nonetheless, additional clinical trials (NCT03185208) are assessing the utility of lithium in preventing AD in elderly patients with mild cognitive impairment.

#### 4.2.2. Parkinson’s Disease

Parkinson’s disease (PD) is the second-most common neurodegenerative disorder and, like AD, tends to occur in older populations. PD is caused by neuronal loss in the substantia nigra, leading to dopamine deficiency, and intracellular accumulation of α-synuclein, called Lewy Bodies (LBs) [125]. Tau pathologies have also been implicated in PD [126]. Early studies demonstrated the localization of GSK-3 in LBs [127,128,129] and noted that GSK-3 polymorphisms, which alter its transcription and splicing, are associated with PD [130]. GSK-3 phosphorylates α-synuclein and tau in PD, which leads to the development of the neurotoxic aggregates that drive the disease [128,131,132,133]. Several inhibitors of GSK-3, including lithium [134,135], indirubin 3′-oxime [136,137], and AR-A014418 [137], have been used in vitro and in animal models to slow neurodegeneration and increase the concentration of dopamine. However, other studies have demonstrated conflicting efficacy of lithium and instead show that it decreases neuronal dopamine levels [138,139]. This underscores a caveat of lithium use in human studies of PD, which is the adverse effect of inducing parkinsonian features [140,141]. This may indicate that the efficacy of GSK-3 inhibition in PD is dose dependent; consistent with this hypothesis, preclinical studies have shown that higher doses of Tideglusib are more neuroprotective [142], though this has not yet been fully recapitulated in human studies of other parkinsonian syndromes [143,144]. Moreover, an ongoing trial (NCT04273932) is evaluating dose titration of lithium in PD.

### 4.3. GSK-3 in Cancer

GSK-3 cannot be strictly characterized as a tumor suppressor or as a proto-oncogene. For example, GSK-3 inhibits the canonical Wnt signaling pathway, functioning in a complex with the classical tumor suppressor adenomatous polyposis coli (APC) to phosphorylate and degrade β-catenin. Loss of *APC* or *GSK3* therefore activates Wnt target genes, such as *MYC* (c-Myc) and *CCND1* (cyclin D1), and promotes tumor growth, as described in greater detail below. GSK-3 also suppresses the epithelial-to-mesenchymal transition (EMT), a critical process in tumor invasion and metastasis (reviewed in [145]) by phosphorylating Snail family transcription factors and targeting them for degradation [146,147,148,149]. While these observations are consistent with a tumor suppressor-like role for GSK-3, GSK-3 also activates NFκB signaling [150,151,152,153], which has a pro-tumorigenic role in the context of inflammatory microenvironments of several cancers (reviewed in [154]). This appears to be through GSK-3 mediated phosphorylation and subsequent stabilization of the adaptor protein NEMO [155], which then promotes NFκB signaling.

**Figure 3:**
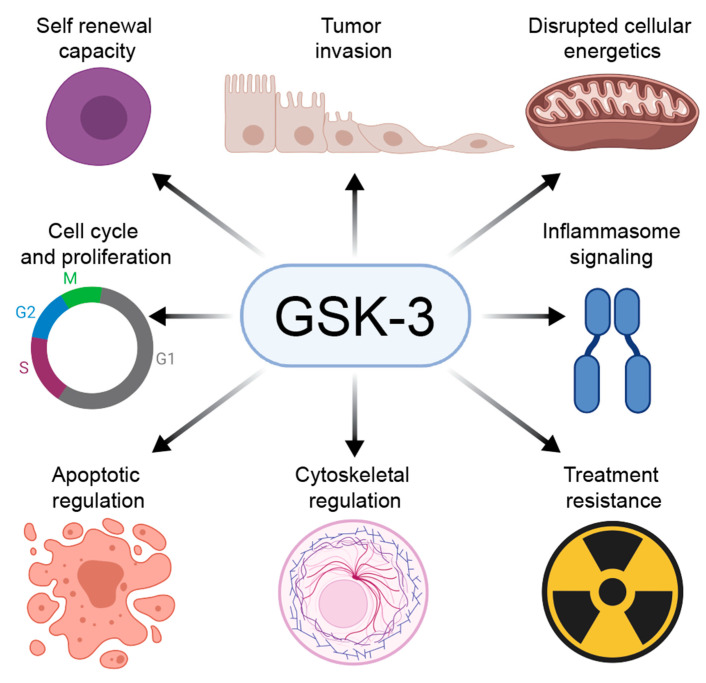
GSK-3 regulates diverse pathways in cancer: GSK-3 can promote or inhibit tumorigenesis by regulating several hallmarks of cancer. These roles vary in the specific contexts of cell type and developmental stage.

Similarly, GSK-3 phosphorylates and destabilizes c-Myc; inhibition of GSK-3 and stabilization of c-Myc can be oncogenic or pro-apoptotic depending on whether c-Myc protein accumulates at intermediate or high levels, respectively [156]. Ultimately, it seems that GSK-3 is tightly regulated in the context of different cell types, developmental stages, and in concert with other signaling molecules; when this regulation is disrupted, it can inhibit or promote malignancy depending on that context (Figure 3). The involvement of GSK-3 in cancer has recently been reviewed in depth [157,158]; therefore, we will provide a detailed mechanistic overview of GSK-3 involvement in certain solid and hematologic malignancies with significant therapeutic potential.

#### 4.3.1. Solid Malignancies

Glioblastoma multiforme (GBM) is the most common malignant brain neoplasm in adults and carries a high mortality. PI3K/AKT signaling, which inhibits GSK-3 activity (reviewed in [159]), is critical in the pathogenesis of GBM [160,161]. However, GSK-3 remains highly expressed and constitutively activated in GBM cells [162,163,164], paradoxically suggesting an oncogenic role for GSK-3 in GBM. Indeed, GSK-3 inhibition through TDZD-8 [165], an allosteric inhibitor of GSK-3 [166], decreased GBM growth and stemness, reflected by impaired neurosphere formation and reduced Nestin levels. Furthermore, both GSK-3 knockdown and its pharmacologic inhibition with LiCl, Kenpaullone, and Enzastaurin impaired GBM proliferation and survival in vitro and in vivo through several mechanisms [167]. First, loss of GSK-3 activity led to transactivation of c-Myc, thereby promoting the expression of pro-apoptotic molecules, including Bim, DR4/DR5, and TRAIL. Second, GSK-3 inhibition altered glucose metabolism and destabilized the mitochondrial membrane, increasing levels of the pro-apoptotic molecule Bax. Third, GSK-3 inhibition impaired NFκB activity, leading to abrogation of pro-survival signals. Although these studies suggest that both GSK-3α and GSK-3β isoforms are oncogenic in GBM, a tumor suppressor role for GSK-3α has also been proposed in GBM by which it promotes apoptotic alternative splice events [168], though this remains incompletely defined and warrants further investigation. Finally, hallmarks of GBM that increase mortality include its invasive nature and propensity for treatment resistance. GSK-3 has been implicated in both of these mechanisms in GBM [169,170,171,172,173]. Therefore, GSK-3 inhibitors have been exploited in pre-clinical studies to improve sensitivity to common GBM treatments, such as temozolomide [174,175], offering a potential therapeutic opportunity.

Colorectal cancer (CRC) is one of the most common cancers in adults. Our understanding of the pathophysiology of CRC has drastically improved survival since it has led to the use of widespread early screening. CRC commonly begins as a precancerous polyp, followed by sequential accumulation of somatic mutations that ultimately lead to malignant transformation; this adenoma-to-carcinoma sequence [176] classically begins with a mutation in the tumor suppressor *APC,* followed by a mutation in the oncogene *KRAS*, and culminates with a mutation of *TP53* [177]. APC is of particular significance in the context of GSK-3 as both are core components of the Axin complex that promotes proteasomal degradation of β-catenin in the absence of Wnt signaling [178]. Loss of *APC* leads to the robust activation of the Wnt pathway frequently observed in CRC [179,180]. APC has several proposed roles in the Axin complex that may contribute to inhibition of Wnt signaling and tumor suppression, including recruitment of β-catenin into the destruction complex, facilitation of ubiquitination and degradation, and direct enhancement of GSK-3 enzymatic activity toward β-catenin [181]. APC enhances GSK-3 activity in vitro and in vivo [181,182], and dissociation of APC from the Axin complex upon Wnt stimulation may explain how Wnt signaling inhibits β-catenin phosphorylation [63,64,65]. Loss of APC and the consequent reduction in GSK-3 activity leads to activation of several downstream targets in addition to β-catenin, including mTOR signaling, MAPK/ERK signaling, and BMP signaling [64].

Despite these compelling data that GSK-3 serves as a tumor suppressor in CRC, the exact role of GSK-3 in this malignancy remains controversial. For example, a conflicting report on the role of GSK-3 in KRAS dependent CRC demonstrated that GSK-3 inhibition decreased cell survival through an increase in β-catenin and c-Myc [183]. Additionally, multiple studies have demonstrated that inhibition of GSK-3 activity may still have therapeutic potential in the management of CRC through the induction of P53 dependent and independent apoptosis [184,185,186,187,188,189], downregulating EMT [190,191,192,193], re-establishing Hedgehog signaling [194], and disrupting malignant cytoskeletal dependencies [190,195,196]. Interestingly, Dow et al. observed that restoration of *Apc* in murine models of CRC triggered intestinal differentiation and tumor regression in spite of activating mutations in *Kras* or inactivating mutations in *Tp53* [197]. However, differentiation occurred prior to full *Apc* expression and did not induce apoptosis. While the authors did not specifically comment on the activity of GSK-3 in this study, their findings underscore the dynamic nature of cell survival and differentiation in CRC; in this regard, a better understanding of how GSK-3 is involved at different stages of tumorigenesis in CRC may help evaluate its efficacy as a therapeutic target.

Despite advances in therapies for other cancers, survival in pancreatic ductal adenocarcinoma (PDAC) remains poor, with incremental improvements through the use of escalating cytotoxic chemotherapy in addition to surgery when possible. Therefore, the possibility of targeted therapy has been of great interest. *KRAS* mutations [198], which occur in nearly 90% of PDAC patients, promote an inflammatory microenvironment that is quintessential to the development of PDAC. In murine models, this occurs through mutant *Kras* induction of IL-1α, which ultimately leads to NFκB activation [199]. This inflammatory state, characterized by high levels of NFκB signaling, can be seen in other preceding risk factors for PDAC, such as chronic pancreatitis (reviewed in [200]). Therefore, GSK-3, which has established relationships with both KRAS and NFκB in other tumors, may have a significant role in the pathogenesis and treatment of PDAC. GSK-3β is highly active in PDAC cells, and both pharmacologic inhibition and genetic ablation of GSK-3 decreased PDAC survival and proliferation [151]. Loss of GSK-3 activity in this model also decreased expression of NFκB target genes involved in proliferation and survival, such as *CCND1*, *BCL2*, *BCL2L1*, and *XIAP*. Subsequent studies have confirmed that sensitivity of PDAC cells to GSK-3 inhibition is in part through loss of GSK-3 dependent NFκB signaling [152,201]. However, GSK-3 inhibition may also impair PDAC survival through other mechanisms such as TRAIL-dependent synthetic lethality [202,203,204] and interruption of pro-survival transcription pathways [205,206,207]. Given the prevalence of chemoresistance in PDAC, several preclinical studies have investigated whether targeting GSK-3 could improve sensitivity to gemcitabine, which is commonly used in PDAC treatment, and have shown promising results, especially in combination with HDAC inhibition [208,209,210]. These findings indicate that GSK-3 inhibitors may ameliorate chemoresistance in PDAC.

Clinically, these findings have prompted several trials of GSK-3 inhibitors in advanced solid tumors. LY2090314 is an ATP-competitive inhibitor of GSK-3 that demonstrated a tolerable safety profile in combination with pemetrexed and carboplatin for advanced/metastatic solid tumors, though a randomized-control trial will be needed to assess the efficacy of this protocol [211]. A Phase I/II trial (NCT016323306) for LY2090314 in metastatic PDAC was unfortunately terminated due to lack of enrollment. Ongoing trials are being performed with agents such as 9-ING-41 in combination with other chemotherapy in adults (NCT03678883) and children (NCT04239092), CHIR99021 to expand NK cells for infusion in patients with various solid tumors (NCT03213964, NCT03319459), and CLOVA (cimetidine, lithium, olanzapine, valproic acid) in combination with gemcitabine for PDAC (UMIN000005095) or temozolomide in GBM (UMIN000005111).

#### 4.3.2. Normal and Malignant Hematopoiesis

Normal hematopoiesis is characterized by a hierarchical evolution of hematopoietic precursors, which undergo several rounds of proliferation followed by either quiescence or lineage determination. GSK-3 has multiple roles in HSC maintenance, as demonstrated by the effects of GSK-3 inhibitors or loss of function in mouse hematopoietic cells. Inhibition of GSK-3 activates Wnt/β-catenin signaling to support HSC self-renewal but also activates mTOR complex 1 (mTORC1) signaling leading to HSC activation and an increase in multipotent progenitors at the expense of self-renewing HSCs [212]. Consistent with the latter findings, activation of mTORC1 [213] and loss of LKB1 [214] promote HSC pool depletion through proliferation of committed progenitors through improved nutrient sensing. Furthermore, combined inhibition of GSK-3 and mTORC1 allows ex vivo maintenance of long term, self-renewing HSCs in a cytokine-independent manner [215]; under these conditions, Wnt activation maintains HSC fate while mTORC1 inhibition prevents differentiation and loss of stemness. HSCs were maintained without loss of long-term renewal capacity for up to one week in the absence of cytokines or stromal cells.

Acute myeloid leukemia (AML), the most common acute leukemia in adults, results from clonal proliferation and maturation arrest of early myeloid progenitors. Progenitors with self-renewal capability have been defined functionally as leukemic stem cells (LSCs) or leukemia initiating cells (LICs) [216]. Wnt/β-catenin signaling has proven to be indispensable for the development of LSCs in AML [217], but several studies have also demonstrated GSK-3 as an important regulator of LSC development and maintenance. LSCs expressing fusion proteins containing Mixed Lineage Leukemia (MLL) have been useful models to study the role of GSK-3 in the development of AML. Wang et al. initially demonstrated that GSK-3 can paradoxically maintain clonal proliferation of MLL-transformed cells and is required for transformation into leukemic cells in murine models through destabilization of p27^Kip1^ [218]. A subsequent study by the same group further elucidated a model in which GSK-3 maintains the association of CREB with MEIS to maintain gene expression profiles associated with HOX mediated leukemic transformation [219]. Although these studies provide strong support for the role of GSK-3 in maintaining LSCs and allowing for transformation, they do not readily explain the paradox of how both GSK-3 and β-catenin are required for LSC development and survival despite the former canonically downregulating the latter. Yeung et al. proposed a model by which β-catenin is progressively activated in the transformation of MLL pre-LSCs to LSCs, and deletion of β-catenin in either subset completely abolished leukemogenicity of MLL-transformed cells [220]. MLL LSCs with high levels of nuclear β-catenin were insensitive to GSK-3 inhibition, while suppression of β-catenin expression re-sensitized cells to GSK-3 inhibition. Therefore, while GSK-3 inhibition may be a suitable treatment in AML or early in the development of LSCs, it may not completely abolish the LSC pool. Moreover, it is likely that GSK-3 inhibition is still effective in treating AML cells through mechanisms outside of maintaining stemness such as NFκB signaling [221,222], modulation of cytotoxic chemosensitivity [223,224], and alterations in apoptotic signaling [225]. It should be noted, however, that inhibition of GSK-3 can also enhance the ex vivo survival of primary leukemic cells from patients with AML [226].

Chronic myeloid leukemia (CML) LSCs also require active Wnt/β-catenin signaling [227]. The Armstrong group hypothesized that this may offer an approach to target minimal residual disease with tyrosine kinase inhibitor (TKI) resistance [228]. In their study, genetic deletion of β-catenin combined with imatinib drastically reduced CML LSC survival, though bulk CML cells persisted. Interestingly, increased β-catenin expression may be a result of aberrant splicing of *GSK3B*, as demonstrated in a study of CML patients, in which CML cells from four of seven patients in blast crisis and one of four in chronic phase CML showed mis-splicing of *GSK3B*, yielding a form that did not interact with AXIN and was unable to phosphorylate β-catenin [229]. However, a clear role for GSK-3 and its inhibition in CML will require further investigation.

The role of GSK-3 in acute lymphoblastic leukemia (ALL), chronic lymphocytic leukemia (CLL), and lymphomas remains poorly understood with primarily correlative pathophysiology driving studies of GSK-3 inhibition in these models. In other tumors, GSK-3 regulates FOXO proteins, PI3K/AKT signaling, NOTCH signaling, and NFAT signaling (reviewed in [157]), all of which are critical in lymphocyte development (reviewed in [230,231]). Despite the lack of in depth mechanistic studies of GSK-3 in normal and malignant lymphopoiesis, preclinical evaluation of GSK-3 inhibitors in ALL [232,233,234], CLL [235], and lymphoma [156,236,237] have been promising, and further investigation is warranted.

In regard to clinical trials, a phase I trial investigating lithium and tretinoin in non-promyelocytic AML demonstrated an acceptable safety profile with observed on-target effects, which led to AML cell differentiation [238]. However, a recent phase II trial in AML found that LY2090314 did not lead to complete or partial remissions despite on-target effects [239]. Therefore, further evaluation of other GSK-3 inhibitors, combination therapies, and an improved understanding of GSK-3 mediated targetable pathways are necessary. Ongoing trials are being performed with CHIR99021 to stimulate NK cell production for infusion in patients with AML (NCT03081780) and with 9-ING-41 in patients with lymphoma (NCT03678883).

## 5. Lithium, GSK-3, and Coronaviruses

### 5.1. Antiviral Activity of Lithium

Potential antiviral activity of lithium has been investigated for over 40 years, as comprehensively reviewed recently [240]. Lithium reportedly antagonizes replication of multiple DNA and RNA viruses in cell culture assays, and in some cases lithium therapy has been associated with modest antiviral activity in patients. While the clinical benefit of lithium and other GSK-3 inhibitors in viral infections is uncertain, the in vitro effects may provide new insights into the roles of host proteins during virus infection. Although inhibition of inositol turnover may also mediate antiviral effects of lithium [240], we will focus here on the potential role of GSK-3 as an antiviral target of lithium, with special attention to the role of GSK-3 in coronavirus infections.

Lithium was reported to have antiviral activity against herpes simplex in the 1970s. These studies included in vitro demonstration that lithium impairs HSV replication in cell culture [240,241,242] and culminated in a series of both retrospective and small prospective clinical trials with lithium carbonate to assess duration and rate of recurrence of labial herpes infections [240,243]. As reviewed by Murru et al., the retrospective study reported a reduced rate of recurrence for labial HSV for patients on lithium carbonate (LiCO3) compared to patients treated for depression, and the protective effect was higher in patients with higher serum lithium levels. Two randomized, placebo-controlled trials with LiCO3 for HSV found a trend towards a modest effect of lithium in reduction of HSV recurrence, but both trials were small and underpowered [240,244].

Lithium has also been reported to have antiviral activity against diverse DNA and RNA viruses in vitro, including hepatitis C, adenoviruses, Dengue virus-2, pseudorabies and vaccinia viruses, parvoviruses, foot-and-mouth disease virus (FMDV), and feline calicivirus (FCV) [240]. Limited studies in patients have also supported modest antiviral effects against HIV and influenza. Prospective studies on HIV were small but reported a transient reduction in viral transcription in HIV infected patients [245]. A retrospective study on 177 patients taking LiCO3 for bipolar disorder also showed a modest but significant reduction in flu-like infections when comparing the incidence in the same patient cohort before and during lithium therapy [246]. There was no effect of treatment in the smaller control group of patients taking antidepressants.

While lithium has direct antiviral activity (described in more detail below for coronaviruses), it may also function indirectly by enhancing host anti-inflammatory responses. The literature on lithium effects on the immune system is complex, with evidence for both pro-inflammatory and anti-inflammatory effects that may contribute to host responses to viral infections, as more fully reviewed in [240]. The effects of lithium and GSK-3 on inflammation are also comprehensively reviewed in [247,248].

### 5.2. Lithium and GSK-3 in Coronaviruses

Perhaps for these reasons, lithium salts have been tested for activity against a number of coronaviruses in cell culture models. LiCl impairs replication and infectivity of diverse coronaviruses, including infectious bronchitis virus (IBV), porcine epidemic diarrhea virus (PEDV), transmissible gastroenteritis virus (TGEV), and the β-coronavirus responsible for severe acute respiratory syndrome (SARS-CoV) [249,250,251,252,253,254,255,256]. These observations have led to recent calls to use lithium to treat COVID19. However, the effects of lithium on these coronaviruses were observed at concentrations well above clinically tolerable levels. Furthermore, experimental evidence to support an inhibitory effect of lithium on SARS-CoV-2, the cause of COVID19, has not yet been reported.

As the IC_50_ for LiCl inhibition of GSK-3 [19,20], as well as the optimal therapeutic plasma concentration in bipolar disorder [20], is ~1 mM, the high concentrations needed to inhibit coronaviruses (at least 5–10 mM) also raise the possibility that lithium may be acting through another target. However, considerable data now support GSK-3 as the direct target responsible for lithium effects on diverse coronaviruses. Initial studies using multiple protein kinase inhibitors hinted that GSK-3 may phosphorylate the nucleocapsid protein [257], and subsequent work from several labs including Yeh and colleagues provided strong support for a critical role for GSK-3 in phosphorylating and enhancing the function of nucleocapsid protein [250,258,259].

Coronaviruses encode a nucleocapsid (N) protein that binds viral genomic RNA (gRNA) and is essential for viral replication, transcription, and virion assembly [250,251,259,260,261]. N is phosphorylated within an arginine-serine (RS) domain that is conserved in coronaviruses [250,257,258,259,262,263,264]. As pointed out by Wu et al., the RS domains in N protein from the JMHV strain of mouse hepatitis virus and SARS-CoV includes the canonical SXXXS motif found in many GSK-3 substrates [250]. N from other coronaviruses diverge in overall sequence but retain RS domains and tandem SXXXS motifs. Evidence that GSK-3 phosphorylates N from JHMV and SARS-CoV includes: (1) GSK-3 binds to N and phosphorylates sites within the SXXXS motifs [250,257,258]; (2) GSK-3 dependent phosphorylation of N from SARS-CoV and JHMV has been documented by mass spectrometry and phospho-specific antibodies [250]; (3) mutation of the +4 priming serine to alanine prevents phosphorylation by GSK-3, blocks N functions, and interferes with viral transcription and replication; (4) siRNA mediated knockdown of both *GSK3* mRNAs (*GSK3A* and *GSK3B*) impairs viral replication and infectivity; (5) inhibition of GSK-3 with Kenpaullone or LiCl impairs replication and infectivity of SARS-CoV and JHMV.

Mechanistically, N phosphorylation plays multiple roles. Immunoprecipitation of N followed by mass spectroscopy shows that, in addition to GSK-3α and GSK-3β, N binds multiple RNA binding proteins involved in splicing and translation [258,259,265]. N phosphorylation is required for the recruitment of the RNA helicase DDX1 to form a complex that mediates transcription of long mRNAs and for replication of genomic RNA [259]; knockdown of *DDX1* or inhibition of GSK-3 impairs recruitment of DDX1, binding of N and DDX1 to viral mRNAs, and viral replication.

Taken together, these observations strongly support a critical role for GSK-3 in N protein function and in coronavirus infections. While the concentrations of lithium required to inhibit these functions of GSK-3 may be too high to treat infected patients, a role for other, clinically well tolerated GSK-3 inhibitors remains a potential option worth further exploration. As GSK-3 is a host kinase that may be required for multiple coronaviruses, targeting it may be effective against diverse pathogenic coronaviruses and this approach may be less susceptible to mutation and evolution of the viral genome. Clinically well tolerated GSK-3 inhibitors include Tideglusib (phase I–II) and Enzastaurin (phase I–III), a PKC inhibitor that also has activity against GSK-3 at a clinically well tolerated concentration [167,266,267,268,269].

Other protein kinases have also been proposed to phosphorylate the RS domain of N, including SR domain protein kinases (SRPK). Like GSK-3, SRPKs tend to processively phosphorylate multiple serines within a local domain. SRPK inhibitors have demonstrated efficacy against Hepatitis C, Sindbis virus, HIV, and cytomegalovirus [270,271] and have been proposed to inhibit coronavirus N protein phosphorylation as well [262].

## 6. Concluding Remarks

Despite the extensive literature on lithium effects, few direct targets have been identified, which is surprising given the simplicity of lithium and its mechanism of inhibition. Thus, the complexity of lithium effects has less to do with the number of targets and more do to with the wide-ranging functions of the known targets, especially GSK-3 and IMPase, which both play fundamental roles in diverse signaling pathways. Here we have focused on GSK-3, which has well over 100 substrates and functions in multiple signaling pathways, including RTK, GPCR, Wnt, and nuclear hormone signaling. GSK-3 manages to be a complicated enzyme even without the many inputs and outputs, as it employs several autofeedback circuits to regulate its own activity. These feedback circuits could play a key role in modulating sensitivity to diverse extracellular signals, for example in postsynaptic neurons. As these regulatory circuits are sensitive to lithium, they may also explain why lithium can ameliorate markedly distinct, pathological mood states without perturbing euthymic mood.

GSK-3 sets itself apart from most signaling kinases by functioning constitutively and, in most cases, inhibiting rather than activating downstream effectors; thus, signaling pathways inhibit GSK-3 to turn on downstream signaling, a recurring double negative meme for GSK-3 signaling. It should not be surprising then that the role of GSK-3 in cancers is complex, with tumor-promoting and tumor-suppressive activities. Even in the well-characterized canonical Wnt signaling pathway, GSK-3 seems to be of two minds, as it is required for activation of the LRP5/6 co-receptor upstream of its more familiar role in suppressing Wnt signaling by driving degradation of β-catenin. With this in mind, it seems likely that the role of GSK-3 in psychiatric disorders and neurodegenerative disorders, as in cancers, will also be complex. We have presented here just an overview of an intricate regulatory system in these disorders, with much left to be uncovered.
